# A New Method for Testing Thermodynamic Consistency of Vapor–Liquid Equilibrium Data

**DOI:** 10.1021/acsomega.5c04650

**Published:** 2025-10-02

**Authors:** Jiří Zbytovský, Tomáš Sommer, Martin Zapletal, Jiří Trejbal

**Affiliations:** Department of Organic Technology, 52735 University of Chemistry and Technology (UCT) Prague, Technická 5, Prague 6 166 28, Czech Republic

## Abstract

Tests of thermodynamic consistency are essential tools for evaluating VLE data quality. However, there is a lack of software that offers the most commonly used testing procedures in a single application. Furthermore, currently used tests are very general and serve well to quantify experimental error but do not reveal much about its cause. In this work, a new test is proposed, called the “gamma offset test”. It is designed to have a focused, limited scope—to detect inconsistency between the binary VLE data set and the corresponding vapor pressure models. The proposed testing procedure was applied to a collection of VLE data sets obtained from the literature, and the results were compared with the tests of Fredenslund and Redlich–Kister. A criterion of consistency to formally accept or reject the data was fine-tuned so that the test provides meaningful results. It was shown that this new test can be a valuable complement to the traditionally used procedures for most binary systems. Moreover, the advantages of the new test were demonstrated in systems where existing procedures are difficult to apply. The new test can be used either to assess experimental setup accuracy or to help diagnose the cause of a known experimental error. This test is part of a newly developed open-source software package called “VLizard, a VLE wizard”, which also offers other well-known testing procedures. With its graphical interface, it aims to fill the gap as an easily accessible tool for both academic and industrial VLE research.

## Graphical abstract

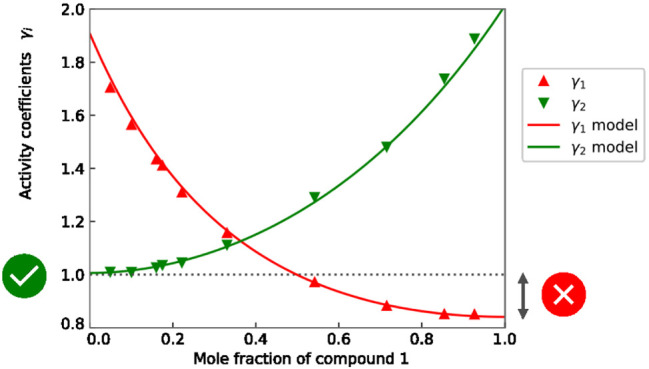

## Introduction

Accurate description of vapor–liquid equilibrium (VLE) is indispensable for the development of chemical industrial technologies, particularly for the design and optimization of unit operations such as distillation, absorption, drying, multiphase reactors, and others. Multicomponent systems, often encountered in the industry, are usually represented by constituent binary systems in order to reduce experimental effort and computational complexity. However, even so, data are often scarce in existing literature, especially when chemical specialties are concerned. If data are available at all, they often have an insufficient range or poor quality. Therefore, experimental laboratory measurement remains indispensable in industrial research.

In order to be used for design and optimization, experimental VLE data are often reduced using thermodynamic models, such as the nonrandom two-liquid (NRTL) model^1^ or the universal quasi-chemical activity coefficient (UNIQUAC) model.^2^ A great deal of effort is often invested in obtaining a good fit with statistical confidence for the estimated parameters. However, that is not enough to guarantee the quality and reliability of the model—the fitted model can only be as good as its source data, which makes VLE data quality evaluation an essential task. If there are preceding data available in the literature, direct comparison with newly measured data presents the simplest and most natural way to assess the quality. However, novel VLE research is usually focused on less-explored systems or different conditions and composition ranges. Measurements of well-known systems are often used to validate the experimental setup. In order to enumerate the VLE data quality in terms of internal consistency rather than external consistency, various authors have proposed tests of thermodynamic consistency.

The binary system is described as a collection of points (*p*, *T*, *x*_1_, *y*_1_), which represent pressure, temperature, and the mole fractions of component 1 in the liquid phase and the vapor phase. Usually, the points are grouped as either isobaric or isothermal data sets, depending on the experimental setup. Additionally, a reliable and accurate vapor pressure (*p* ^◦^) model for each pure component is indispensable for describing the system.

Most of the testing procedures are based on the Gibbs–Duhem equation and rely on the fact that the measured data, as described above, are overdetermined. The tests then examine the internal consistency of the data with the Gibbs–Duhem equation and quantify the deviation from it. The Gibbs–Duhem equation is impractical for direct applications, being a partial differential equation, and also because it requires knowledge of excess volume and enthalpy of mixing—data that are not always available. Therefore, various authors have proposed approaches to simplify it mathematically. The most well-known testing procedures include the “area test”, for example, by Redlich–Kister^3^ or by Herington,^4^ and several deviation tests, such as the Fredenslund test,^5^ the van Ness point-to-point test,^6^ the Kojima test,^7^ and the Kang test.^8^ The slope test^9^ is also worth mentioning for its historical importance. It should be noted that all the procedures cited and discussed in this article are designed only for binary systems, assuming nonideality in the liquid phase, albeit with only a single phase. Very few procedures exist to cover other cases, an example being the McDermott–Ellis test, which covers ternary systems.^10^ There is also novel research published by Fernández et al.^11–13^ proposing testing procedures even for systems with two liquid phases (VLLE or LLE), which are even more mathematically challenging to test.

Performing the tests is often required for the academic publication of measured VLE data. However, this requirement should not be considered merely a formal one. The tests should not be omitted for commercial applications either, as an unreliable thermodynamic VLE model could have a significant impact on process design and economic performance. Multiple testing procedures should always be applied to give a broader view of the data quality, because each procedure has different limitations due to the approximations used in their theoretical basis.^9^ This presents a time-consuming task, as there is a lack of software that offers a comprehensive set of tests in a user-friendly manner. The original literature sources sometimes include source code appendices (e.g.,^5^), but due to their age, advanced knowledge of programming may be needed to apply them effectively. Commercial process simulation software packages such as Aspen Plus^14^ include only some of the tests, and they are not freely available. A software package that implements all of the most common testing procedures with a unified interface could therefore prove to be a valuable tool for both academic and industrial research. This article aims to fill the gap by presenting new free open-source software called “VLizard, a VLE wizard”.

Furthermore, the testing procedures mentioned above are all designed to detect measurement error in the data and to quantify it, but not much is revealed about the cause of the error. A new testing procedure called the “gamma offset test”, or γ offset test for short, is proposed in this article. This new test is a development of the “end point test” devised by Smith et al.,^15^ and it is deliberately designed with limited scope to help pinpoint one of the common causes of thermodynamic inconsistency. Such a procedure could become an auxiliary tool for researchers to use alongside well-known testing procedures.

## New Testing Procedure

### Core Concepts of the New Test

Vapor–liquid equilibrium conditions are fully described by the Raoult–Dalton law^16^ as per eq 1, where $p_{i}^{◦}$ is the vapor pressure of pure component *i* at temperature *T*, ϕ is the fugacity coefficient of the mixture vapor phase, and $\phi_{i}^{◦}$ is the fugacity coefficient of pure component *i* saturated vapors.

1 $$y_{i}\phi p=\gamma_{i}x_{i}\phi_{i}^{◦}p_{i}^{◦}$$

Let us consider a pure component: *x*_*i*_ = *y*_*i*_ = 1. At its boiling point, the system pressure *p* is identical to $p_{i}^{◦}$ , and likewise, *ϕ* is identical to $\phi_{i}^{◦}$ . eq 1 then reduces to *γ*_*i*_ = 1. This fact is trivial for pure components as it stems from the activity coefficient definition. However, this concept can also be applied to a binary mixture. Let us define *K*_*i*_, the overall nonideality factor of component *i*. Its definition is given in eq 2, while its practical calculation from experimental data is given in eq 3. For a binary mixture, we can state that *K*_*i*_ must converge to 1 as the composition approaches that of a pure component. This is formally expressed in eq 4 and represents the core concept of the proposed test.

2 $$K_{i}=\frac{\gamma_{i}\phi_{i}^{◦}}{\phi }$$

3 $$K_{i}^{\exp}=\frac{y_{i}p}{x_{i}p_{i}^{◦}}$$

4 $$\underset{x_{i}\to 1}{\lim}K_{i}=\underset{x_{i}\to 1}{\lim}\gamma_{i}=1$$

In case $K_{i}^{\exp}\left(x_{i}=1\right)$ , extrapolated from the experimental data, deviates from 1, it is a clear indication of a mismatch between VLE data and the used $p_{i}^{◦}$ models. Either of them may be the source of the error. Vapor pressure models for pure components may be obtained from the literature, for example, in the form of Antoine^17^ or Wagner^18^ equations. Or better yet, the model may be fitted to vapor pressure data measured on the same apparatus as the VLE data, which is all the more reason to expect perfect agreement.

Note that at the *x*_*i*_ = 1 limit, *K*_*i*_ reduces to *γ*_*i*_, and this will be referred to as such for better clarity and familiarity.

### Vapor-Phase Nonideal Behavior Modeling

Ideal gas behavior is a common assumption made by many experimental authors cited in this work, which simplifies eq 1 by setting $\phi =\phi_{i}^{◦}=1$ . It may be sufficient for gaseous mixtures with weak interactions between molecules or for data measured at lower pressures or high temperatures, but that is often not the case. Many polar species are strongly associated in the vapor phase even at lower pressures, for example, formic and acetic acid^19,20^ which form hydrogen bonds. On the other hand, low-boiling components are often measured at very high pressures and/or low temperatures, also yielding significant deviations from ideal gas behavior.

In this work, the first-order virial equation is used to model the gas compressibility factor *Z* as per eq 5.^16^ Because the molar volume *V*_*m*_ is typically not directly measured, calculating it from experimental data would yield a transcendental equation. As a simplification, only in the context of the virial equation itself, *V*_*m*_ will be approximated using the ideal gas law, where *R* is the universal gas constant. As will be discussed later, the model is used solely as an empirical fitting, so this slight deviation from rigorous thermodynamic definitions is acceptable.

5 $$Z=1+\frac{B}{V_{m}}\approx 1+B\frac{p}{RT}$$

The fugacity coefficient is calculated as per eq 6,^16^ where *P* is a pressure integration variable.

6 $$\ln\phi =\int_{0}^{p}\frac{Z-1}{P}dP\approx \int_{0}^{p}\frac{B}{RT}dP=\frac{Bp}{RT}$$

A common approach for modeling a gas mixture is to consider it a single pseudocomponent, whose properties are a function of composition, as per eq 7.^16^ Its virial coefficient *B* is then calculated from the coefficients for both pure components *B*_*i*_ and the symmetrical cross coefficient *B*_12_.

7 $$B=y_{1}^{2}B_{1}+2y_{1}y_{2}B_{12}+y_{2}^{2}B_{2}$$

### The New Test Procedure

The Gibbs–Duhem equation, which is used for conventional thermodynamic consistency tests, requires calculating the activity coefficient *γ*_*i*_ for each data point. This is often one of the first steps when processing experimental VLE data. As mentioned above, the inconsistency between the VLE data and the *p* ^◦^ models presents a systematic error. Researchers may visualize *γ*_1_(*x*_1_) and *γ*_2_(*x*_1_) plots to assess it intuitively, but as of today, there is no conventional procedure to quantify the deviation and formally accept or reject the data based on a formal criterion. The *γ* offset test is therefore proposed to address this need. It should be noted that the test is not based on the Gibbs–Duhem equation like traditional testing procedures but rather on compliance with basic properties of the activity coefficient stemming from its definition.

As mentioned above, the binary mixture should converge to the pure component behavior at the pure component limit. To verify this for a single data set, which is either isobaric or isothermal, the data set can be fitted with a *γ*_*i*_(*x*_*i*_, *T*) model, which is then extrapolated to *x*_*i*_ = 1. The proposed procedure is based on the broadly used NRTL model^1^ as described by the set of eq 8.

8 $$\begin{array}{ll} \ln\gamma_{1}^{\mathrm{NRTL}} & =x_{2}^{2}\left(\tau_{21}{\left(\frac{G_{21}}{x_{1}+x_{2}G_{21}}\right)}^{2}+\frac{\tau_{12}G_{12}}{{\left(x_{2}+x_{1}G_{12}\right)}^{2}}\right)\\ \ln\gamma_{2}^{\mathrm{NRTL}} & =x_{1}^{2}\left(\tau_{12}{\left(\frac{G_{12}}{x_{2}+x_{1}G_{12}}\right)}^{2}+\frac{\tau_{21}G_{21}}{{\left(x_{1}+x_{2}G_{21}\right)}^{2}}\right)\\ \ln G_{ij} & =-\alpha_{ij}\tau_{ij}\\ \tau_{ij} & =a_{ij}+b_{ij}/T\\ \alpha_{ij} & =c_{12} \end{array}$$

The NRTL model is modified to include two additional parameters, *E*_1_ and *E*_2_ (the error parameters), as shown in eq 9.

9 $$\begin{array}{ll} \gamma_{1}^{\mathrm{calc}} & =\gamma_{1}^{\mathrm{NRTL}}+E_{1}\\ \gamma_{2}^{\mathrm{calc}} & =\gamma_{2}^{\mathrm{NRTL}}+E_{2} \end{array}$$

We may use the virial equation to account for gas-phase nonideality. The overall nonideality coefficient *K*_*i*_ model is composed of eqs 2, 9, 6, and 7. Meanwhile, the experimental data are processed into $K_{i}^{\exp}$ values as per eq 3. It should be noted that as experimental researchers, we only assess the overall nonideal deviation of the VLE system. We do not know in advance to what extent it can be ascribed to the vapor versus liquid phase unless we have access to gas-phase equation of state measurements.

Nonlinear regression is then used to determine the parameters *a*_12_, *a*_21_, *b*_12_, *b*_21_, *b*_1_, *B*_12_, *B*_2_, *E*_1_, *E*_2_. The NRTL nonrandomness parameter *c*_12_ is excluded from optimization and fixed at a chosen value, as described by the original authors.^1^ If an ideal gas is assumed, the virial parameters *B*_1_, *B*_12_, and *B*_2_ shall be fixed at 0.

Because extrapolation to *x*_*i*_ = 1 is of utmost importance, the optimization objective function *f* is chosen accordingly. It is based on the root-mean-square error and is defined in eq 10. The difference between *K* calculated from the model (calc) and from measured data (exp) is calculated as *r*_*i,j*_ for the *j*-th data point of the *i*-th component for all *n* data points. Residuals for both components are added together, but each is weighted by the liquid-phase mole fraction of the same component. This means the fitting is done with special consideration for near-pure regions for both *K*_1_ and *K*_2_, in order to increase the reliability of the *K*_*i*_(*x*_*i*_ = 1) and *γ*_i_(*x*_*i*_ = 1) extrapolation.

10 $$\begin{array}{ll} f\left(a_{12},a_{21},b_{12},b_{21},B_{1},B_{12},B_{2},E_{1},E_{2}\right) & =\sqrt{\frac{1}{n}\overset{n}{\underset{j=1}{\sum }}\overset{2}{\underset{i=1}{\sum }}{\left(x_{i,j}r_{i,j}\right)}^{2}}\\ r_{i,j} & =K_{i,j}^{\mathrm{calc}}-K_{i,j}^{\exp} \end{array}$$

Finally, we obtain the absolute deviation of activity coefficients from 1 extrapolated at *x*_*i*_ = 1. We denote it as Δ*γ*_*i*_, and it is identical to *E*_*i*_. In absolute value, we may call it the “gamma offset”. When both |Δ*γ*_1_| and |Δ*γ*_2_| do not exceed the consistency criterion Δ_max_, the experimental data set may be declared consistent with the *p* ^◦^ models. Otherwise, it is declared inconsistent, pointing toward a significant source of error within the data set.

It should be noted that in this procedure, the combination of the NRTL model with the first-order virial equation is essentially used as an empirical fitting equation. Let us emphasize that the enumeration of *E*_1_ and *E*_2_ is the prime objective of the whole procedure. Any equation could be used as the basis, provided it naturally extrapolates *K*_*i*_(*x*_*i*_ = 1) = 1 without the *E*_*i*_ terms. However, the NRTL model was chosen for its excellent theoretical background on VLE liquid-phase nonideality. Since it has temperature-dependent terms, it is suitable for isobaric data sets as well as isothermal. Meanwhile, the virial equation is the simplest way to model the nonideality of the gas phase, including mixing effects. Given its thermodynamic background, this combined model is expected to provide a more reliable fit than purely empirical numerical models. This model is therefore expected to provide a good fit for a wide range of binary systems, while offering a reasonable number of parameters to optimize. If the data set is particularly small and overfitting is a concern, the virial equation may be excluded, followed by the NRTL parameters *b*_12_, *b*_21_, which may also be fixed at 0. In this minimalistic form, only 4 parameters remain to optimize. Note that the temperature-dependent terms for the isobaric data are rather simplistic in the given form. More parameters might be needed for a good NRTL fit across a wide range of temperatures (several data sets). However, in the proposed test, a separate NRTL model optimization is performed for each data set, meaning that the temperature range is at most that of a single isobaric data set.

Other models were considered for a similar modification: the UNIQUAC model^2^ was dismissed for its high number of parameters. The van Laar model, being an isothermal model, was dismissed because of its unsuitability for isobaric data. It would also be problematic to use it with strongly nonideal systems. Meanwhile, models based on infinite dilution were also considered, but the point of the test is to evaluate the consistency of the data set as a whole—instead of focusing the model on the near-pure regions, a reliable description of the entire composition range is used to extrapolate to *x*_*i*_ = 1.

It must be noted, though, that a VLE data set may not cover the whole composition range uniformly. If one of the *x*_*i*_ ≈ 1 regions is not populated by data points, the optimized *E*_*i*_ parameter will not be reliable. The test can then provide only a partial result—consistency of the VLE data with only one of the two $p_{i}^{◦}$ models.

The new procedure is comparable to the endpoint test devised by Smith et al,.^15^ which is also focused on detecting mismatches between VLE data and *p* ^◦^ models. In their work, the authors proposed the direct extrapolation of pressure and temperature to the pure component limit. The extrapolated *p*(*x*_*i*_ = 1) and *T*(*x*_*i*_ = 1) values are then compared with the calculated pure component vapor pressure and boiling point, respectively. The endpoint test is a simpler, more direct approach—without any further data reduction; only one quantity at a time is directly numerically extrapolated. We believe that the newly proposed “gamma offset test” offers two major advantages. First, the whole available data as *p*,*T*,*x*_1_,*y*_1_ is reduced into *K*_*i*_, respectively γ_*i*_, reducing the influence of random errors in a single quantity. Second, the extrapolation is done after fitting the data with a thermodynamically based model instead of a purely empirical approach.

Let us also remark that the researcher should determine the Δ_exp_γ uncertainty calculated from known experimental uncertainties Δ_exp_ *p*, Δ_exp_ *T*, Δ_exp_ *x*_*i*_, and Δ_exp_ *y*_*i*_. Depending on the experimental setup, the uncertainty may be significant. In the case of Δ_exp_ *γ* ≥ |Δ*γ_i_* |, nothing can be concluded about the consistency of the data set.

### VLizard Software Package

The newly developed testing procedure, as well as several well-known existing testing procedures, is implemented in the “VLizard” application, which is open-source and freely available.^21^ The desktop application is centered around the core program written in Python, which handles the calculations and visualization. The core program is built on the linear algebra framework NumPy,^22^ uses numerical routines offered by the SciPy^23^ library, and visualizes results using the Matplotlib^24^ library. The application also features a graphical user interface written in TypeScript, which offers unified input and output for various testing procedures. It is designed to be user-friendly and intuitive, with the aim of being used by researchers with no programming background.

The main use case for a researcher can be described as follows: First, the user creates a binary system and pastes the experimental VLE data into the application as one table per data set. All entered data are persistent in the application. Next, the *p* ^◦^ models for both components are needed as a prerequisite for further calculations. These can be defined using either Antoine^17^ or Wagner^18^ equations. The Antoine equation is also offered in an extended 7-parameter form, as per the definition from the Aspen Plus V14.5 manual,^14^ which is written in eq 11. If the user only has experimental *p*,*T* data in lieu of model parameters, the program can perform nonlinear regression on the data to optimize a model of choice. For that, the program first uses the trust-region reflective algorithm^25^ to obtain the initial estimate of parameters, considering only errors in *T* as the dependent variable. The parameters are then refined using the orthogonal distance regression algorithm,^26^ which takes into account errors in both *p* and *T*.

11 $$\ln p=A+\frac{B}{T+C}+DT+E\ln T+FT^{G}$$

Having both VLE data and *p* ^◦^ models available, several options become available. The user may visualize the system VLE, which is represented by *x*-*y* and γ_*i*_ plots, a *T*-*x*-*y* plot for isobaric data, or *p*-*x*-*y* plot for isothermal data. Coefficients of variation for *p* and *T* are compared to automatically detect whether the system is isobaric or isothermal. Most importantly, the testing procedures may now be used. The Fredenslund test is implemented as per^5^ and uses the Levenberg–Marquardt algorithm^27^ for nonlinear regression to obtain the Gibbs excess energy model parameters. These parameters represent a linear combination of Legendre polynomials of order *n*_L_, which can be chosen between 3 and 5, with 4 being the default value (as recommended by the test authors). The Gibbs excess energy model is used to calculate the pressure *p* ^calc^ and vapor-phase mole fractions $y_{i}^{\mathrm{calc}}$ , which are then compared to the experimental data. Visualization of the residuals is available, which is useful for assessing each point individually. The results are then aggregated for the entire data set as the average relative deviation of pressure $\overset{―}{\delta p}$ and the average absolute deviation of vapor-phase mole fractions $\overset{―}{\Delta y_{i}}$ . These are defined by eqs 12 and 13, where *N* is the number of data points.

12 $$\overset{―}{\delta p}=\overset{N}{\underset{j=1}{\sum }}\frac{1}{N}\frac{\left|p_{j}^{\exp}-p_{j}^{\mathrm{calc}}\right|}{p_{j}^{\exp}}$$

13 $$\overset{―}{\Delta y_{i}}=\overset{N}{\underset{j=1}{\sum }}\frac{1}{N}\left|y_{i,j}^{\exp}-y_{i,j}^{\mathrm{calc}}\right|$$

Both area tests, which are implemented as per,^3,4^ rely on numerical integration to obtain the two areas *a* and *b*, as shown in eq 14. Due to the need to extrapolate the data to pure components, the integrated function is first fitted with a spline.^28^ The extrapolated function is then integrated from 0 to 1 using the Quadpack^29^ library. The metric *D*, shared by both tests, is given in eq 14, while the metric *J*, specific to the Herington test, is defined in eq 15. Note that the Herington test was designed for isobaric data, so *J* yields 0 for isothermal data sets, and the test becomes identical to that of Redlich–Kister.

14 $$D=100\frac{\left|a-b\right|}{a+b}=100\frac{\left|\int_{0}^{1}\ln\frac{\gamma_{1}}{\gamma_{2}}dx_{1}\right|}{\int_{0}^{1}\left|\ln\frac{\gamma_{1}}{\gamma_{2}}\right|dx_{1}}$$

15 $$J=150\frac{T_{\max}-T_{\min}}{T_{\min}}$$

The slope test is seldom used due to the unreliability of numerical derivation,^9^ as the data are often sparse and encumbered with errors. Nevertheless, VLizard offers an implementation based on a three-point nonequidistant derivation formula. Three points are chosen as a compromise between random error sensitivity and specificity to identify points suspicious of gross error. For each point *j*, the residual *r*_*j*_ is calculated as per eq 16, which should be close to 0.

16 $$r_{j}=x_{1,j}\frac{d\ln\gamma_{1,j}}{dx_{1}}+x_{2,j}\frac{d\ln\gamma_{2,j}}{dx_{1}}$$

Naturally, VLizard also implements the above-described *γ* offset test. The Levenberg–Marquardt algorithm^27^ is used to fit the modified NRTL and virial model. In case the virial equation is requested, the optimization is done in two steps for better numerical stability: first, it optimizes only the NRTL model parameters, which are then used as an initial estimate for optimization with the virial equation included. The program also implements some safeguards against overfitting by automatically excluding parameters. For isothermal data, NRTL *b*_*ij*_ parameters are excluded, as temperature-dependent terms do not add any value there. If the virial equation is requested but the number of points is less than or equal to the number of parameters, the virial equation is disabled. In order to use the virial equation, at least 10 data points are required for isobaric data (9 parameters) and at least 8 for isothermal data (7 parameters). If the number of points is still insufficient, the *b*_*ij*_ parameters are excluded even for isobaric data, reducing the number of parameters to merely 4. Without the temperature-dependent terms, the NRTL model may no longer be suitable for isobaric data. In any case, performing the γ offset test with fewer than 5 data points is discouraged. The program allows the procedure to be executed with 4 data points as a minimum, issuing a warning. The procedure was performed on a multitude of data sets, and the results are examined and discussed below.

Last but not least, the program offers the van Ness point-to-point test, which is implemented as described.^6^ This involves calculating the residuals between $\ln\frac{\gamma_{1}}{\gamma_{2}}$ calculated from the experimental data and a known thermodynamic model. The residuals are then aggregated as root-mean-square values. Deploying this test, therefore, requires prior knowledge of an activity coefficient model. That is why VLizard offers several built-in models, namely those of van Laar,^16^ Margules,^16^ the NRTL model,^1^ and the UNIQUAC model.^2^ Besides the basic definitions from original sources, extended formulas are also provided for the latter two, with 10 and 13 parameters respectively, as defined in the Aspen Plus V14.5 manual.^14^ As in the case of *p* ^◦^ model regression, the user may choose between entering known model parameters and performing nonlinear regression using the Levenberg–Marquardt algorithm.^27^ The regression considers errors in *γ*_1_ and *γ*_2_ as dependent variables. The model fitted from a selected subset of the binary system data can then be used for the van Ness test or to tabulate and visualize *x*-*y*, *T*-*x*-*y*, *p*-*x*-*y,* and *γ_i_* plots at a given pressure or temperature. MINPACK^30^ is utilized to calculate the boiling point at a given pressure, both for pure components from the *p* ^◦^ models or for binary mixtures from the fitted VLE model.

### Results and Discussion

Most of the functions provided by the VLizard software are implementations of procedures already described in the literature, but the “gamma offset test” is a novel contribution.

demonstrates all the above-described procedures on two methanol–water data sets, labeled “A”^31^ and “B”.^32^ shows the Fredenslund test aggregated results, while show its results for individual data points. The integration procedure, which is shared by both of the area tests, is visualized in . Final results of the area tests (*D* for the Redlich–Kister test and |*D*-*J*| for the Herington test) are summarized in . The definitions of *D* and *J* are given in eqs 14 and 15. Example slope test residuals are plotted in for individual data points. summarizes the NRTL parameter optimization results. then displays the *γ_i_* values calculated from the two fitted models, along with the *γ*_*i*_ values for each data point. Using the respective NRTL models for both example data sets, the van Ness point-to-point test results are displayed in then shows the van Ness test residuals for individual data points.

But the main focus of this work remains the new “gamma offset test”, which is designed to detect discrepancies between VLE data and the *p* ^◦^ models. It is expected that data with errors of such kind will not comply with the Gibbs–Duhem equation and will be rejected by other testing procedures. The Fredenslund test is chosen as a reference, as it has a solid theoretical basis and is often regarded highly in the literature.^9^ Rejection of data by the *γ* offset test is expected to imply rejection of the data set by the Fredenslund test. The proposed test quantifies and formalizes a decision process that researchers may already be performing qualitatively and intuitively. The problem can be illustrated with two methanol–water data sets. Figure 1 shows data from Kurihara et al.,^31^ labeled “A”, that seem to satisfy γ_i_(*x*_1_ = 1) ≈ 1 quite well. On the other hand, Figure 2 shows data from Bredig et al.,^32^ labeled “B”, that exhibit an easily noticeable deviation. Table 1 lists the numerical results as per the procedure described above, where *c*_12_ was fixed at 0.3, and the virial equation was not used. Note that the first component named in the system (in this case, methanol) is assigned index 1 in this work.

**Figure 1:**
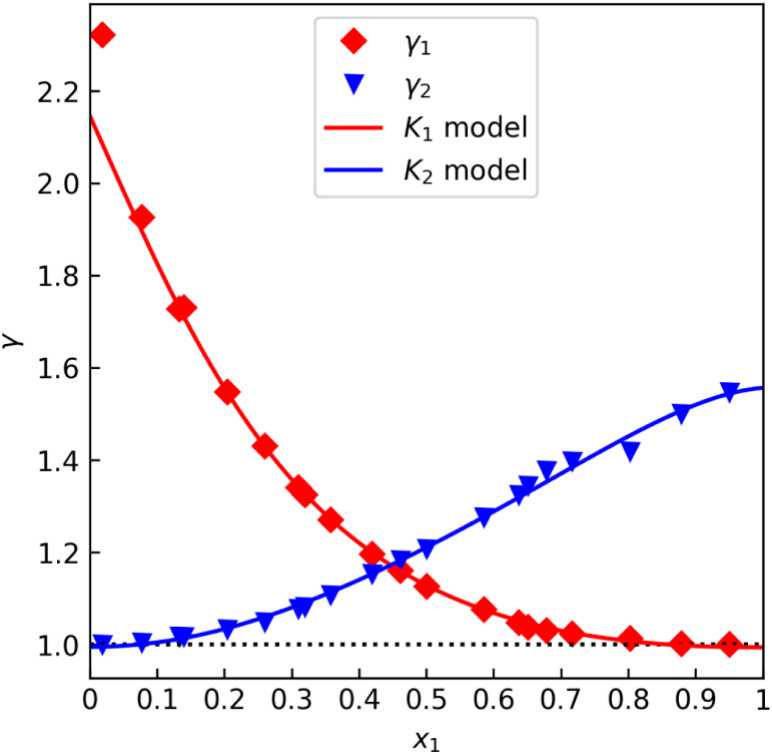
*γ* offset test for the methanol–water data set “A” by Kurihara et al.^31^ (isobaric at 101.3 kPa).

**Figure 2:**
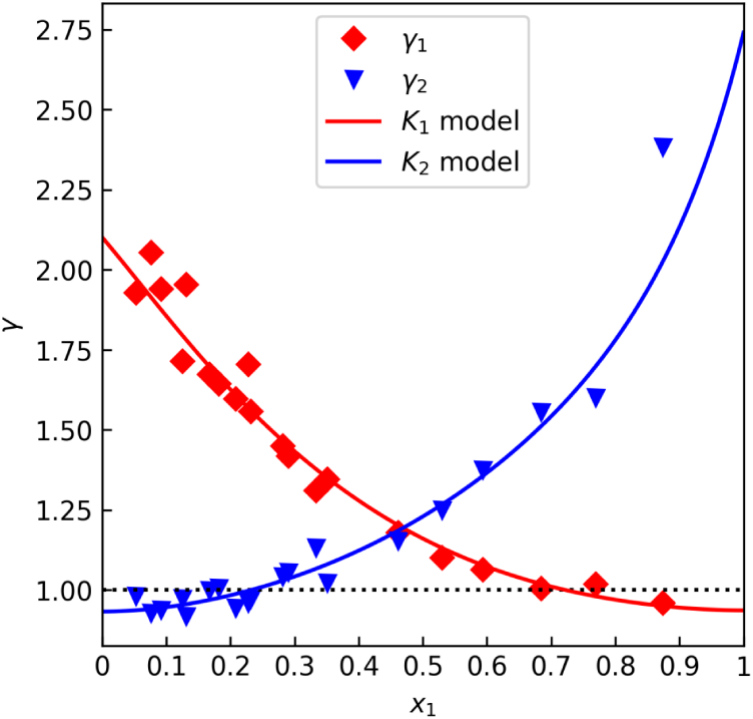
*γ* offset test for the methanol–water data set “B” by Bredig et al.^32^ (isobaric at 101.3 kPa).

**Table 1:** *γ* Offset Test Detailed Results for Methanol–Water Data Sets “A” by Kurihara et al.^31^ and “B” by Bredig et al.^32^ ^a^

Source	*E*_1_	*E*_2_	*γ*_1_(*x*_1_ = 1)	*γ*_2_(*x*_2_ = 1)	|Δ*γ*_1_|	|Δ*γ*_2_|
A^31^	–0.00569	–0.00466	0.994	0.995	0.6%	0.5%
B^32^	–0.0631	–0.0668	0.937	0.933	6.3%	6.7%

a Both data sets are isobaric at 101.3 kPa. The *E*_1_ and *E*_2_ parameters are defined in eq 9. The *c*_12_ parameter was fixed at 0.3, and the ideal gas equation was used.

A criterion of consistency Δ_max_ must be set so that the expected implication of thermodynamic consistency rejection is fulfilled as closely as possible. This means finding a value high enough to eliminate false negative results (rejecting thermodynamically consistent data) and, with lesser importance, low enough so that false positive results occur less frequently (accepting thermodynamically inconsistent data). Moreover, the test should be as general as possible, i.e., applicable to as broad a range of systems as possible, not just particular ones. For that purpose, a number of binary systems were chosen to cover a broad range of boiling points and diverse ranges of chemical properties. These include nonpolar hydrocarbons, polar organic compounds in aqueous or anhydrous mixtures, and ionic species as well. Correspondingly, the selection represents systems with behavior close to ideal as well as strongly nonideal systems. At the given pressures, some of these systems exhibit azeotropic behavior, but they always form only one liquid phase (to conform to the limitations of this methodology). The selection includes atmospheric pressure experimental data, high-boiling components measured at vacuum pressures, and gases liquefied at high pressures and/or cryogenic temperatures. Some of these are well-known systems that have been studied extensively, while others are specialty chemicals with very few published data.

### Comparison of Test Results

In Table 2, the results of the γ offset test are compared with tests by Fredenslund and Redlich–Kister for 52 selected VLE data sets from the literature references. The order of Legendre polynomials for the Fredenslund test was always set as *n*_L_ = 4. The NRTL *c*_12_ parameter was fixed at 0.3. The Herington test was not included in the comparison because it is not applicable to isothermal data sets. The van Ness point-to-point test was not included either, as it concerns the consistency of the VLE data with a known activity coefficient model of choice rather than internal consistency. The slope test was also excluded due to its numerical instability and poor reliability, as discussed in the literature.^9^

**Table 2:** Comparison of Results of the *γ* Offset Test with Fredenslund and Redlich–Kister Tests for Selected VLE Data Sets^a^

System	Data set label and source	Constant condition	*γ* offset ideal gas max(|Δ*γ*_*i*_|)	*γ* offset virial eq max(|Δ*γ*_*i*_|)	Fredenslund max $\left(\overset{―}{\delta p},\overset{―}{\Delta y_{i}}\right)$	Redlich–Kister *D*
MeOH–H_2_O	A^31^	101.3 kPa	0.60%	0.60%	0.96%	5.7
	A*^31^	101.3 kPa	0.60%	1.00%	0.86%	4.8
	B^32^	101.3 kPa	6.70%	20.80%	4.67%	16.0
	C^33^	37.6 kPa	0.70%	0.40%	2.29%	1.7
	D^34^	101.3 kPa	0.80%	4.10%	1.71%	11.0
EtOH–H_2_O	A^31^	101.3 kPa	1.10%	2.50%	1.12%	3.0
	B^35^	32.9 kPa	1.20%	1.60%	1.11%	3.8
	B*^35^	32.9 kPa	1.60%	2.10%	1.38%	5.9
	C^36^	101.3 kPa	1.80%	1.10%	3.23%	4.2
	D^37^	101.3 kPa	0.90%	0.50%	0.89%	1.7
Hex–Hept	A^38^	94.0 kPa	0.70%	1.50%	0.89%	99.1
	B^39^	101.0 kPa	0.70%	1.20%	0.98%	94.3
BZN–TOL	A^40^	101.3 kPa	0.20%	0.50%	0.28%	28.4
	B^41^	101.3 kPa	0.50%	0.30%	0.18%	2.5
	C^42^	101.0 kPa	0.30%	0.70%	1.45%	100.0
	D^43^	101.3 kPa	1.20%	3.10%	1.31%	100.0
Hept–TOL	A^44^	101.0 kPa	1.20%	3.90%	0.61%	12.3
	B^45^	101.0 kPa	0.30%	N/A	0.42%	13.7
	C^46^	101.0 kPa	1.60%	3.00%	1.21%	31.9
	D^47^	6.7 kPa	1.70%	2.60%	1.17%	8.1
	E^48^	101.0 kPa	1.10%	9.20%	1.06%	15.8
CPOL–CPF	A*^49^	10.0 kPa	0.60%	0.40%	1.10%	7.5
	B*^49^	25.0 kPa	0.90%	0.40%	1.40%	9.6
	C*^49^	40.0 kPa	0.60%	2.60%	0.57%	7.8
CHOL–CHF	A*^49^	9.9 kPa	1.30%	0.90%	0.63%	5.3
	B*^49^	25.1 kPa	0.80%	0.60%	0.55%	9.7
	C*^49^	40.0 kPa	0.70%	0.90%	0.20%	6.7
MeOH–THF	A^50^	325.1 K	12.80%	12.30%	2.40%	33.5
	A*^50^	325.1 K	12.70%	12.30%	2.24%	15.0
	B^51^	101.3 kPa	1.40%	3.50%	0.52%	3.0
Pin–Lim	A^52^	323.1 K	13.10%	12.70%	3.25%	69.5
	A*^52^	323.1 K	13.50%	13.10%	3.46%	72.8
	B^53^	101.3 kPa	1.10%	1.30%	0.88%	100
DEA–H_2_O	A^54^	322.2 K	4.50%	6.20%	0.74%	0.6
	B^54^	329.9 K	5.00%	9.20%	1.61%	7.9
	C^55^	101.3 kPa	9.10%	11.00%	23.90%	30.6
HCOOH–H_2_O	A^56^	101.3 kPa	1.00%	2.30%	0.89%	34.3
	B^57^	101.0 kPa	1.90%	2.80%	1.49%	9.5
	C^58^	101.0 kPa	2.10%	3.10%	2.01%	11.9
	D^59^	99.3 kPa	7.50%	N/A	1.91%	45.6
H_2_O–AcOH	A^60^	26.7 kPa	4.70%	4.50%	2.80%	19.9
	B^61^	101.0 kPa	1.70%	1.00%	2.51%	60.5
	C^62^	100.0 kPa	2.70%	1.50%	2.19%	16.1
Prop–iBut	A^63^	293.1 K	4.30%	1.40%	5.25%	100.0
	B^64^	338.7 K	6.90%	0.80%	6.23%	100.0
	C^65^	249.2 K	3.50%	11.90%	5.69%	95.9
O_2_–N_2_	A^66^	100.1 K	3.00%	3.90%	3.92%	90.3
	B^67^	1.824 MPa	3.40%	13.20%	6.80%	100.0
Palm–Stear	A^68^	6.67 kPa	3.90%	N/A	1.26%	50.3
HCOOEt–Hex	A*^69^	101.3 kPa	2.00%	1.10%	1.92%	1.3
HCOOEt–Hept	A*^69^	101.3 kPa	1.30%	0.50%	6.03%	15.7
HCOOEt–Okt	A*^69^	101.3 kPa	6.00%	2.50%	12.60%	25.9
HCOOEt–Non	A*^69^	101.3 kPa	3.70%	0.40%	24.70%	42.2
HCOOEt–Dec	A*^69^	101.3 kPa	7.60%	2.10%	64.20%	70.6
FA–Isop	A*^70^	373.1 K	48.10%	N/A	2.65%	59.9
	B*^70^	393.1 K	54.90%	N/A	9.1 9%	69.8

a The asterisk sign means that the *p* ^◦^ models used were the ones published or referenced in the same source. Otherwise, extended Antoine equation parameters from the Aspen Plus V14.5 software were used.

The binary systems in question are methanol–water (MeOH–H_2_O), ethanol–water (EtOH–H_2_O), propane–isobutane (Prop–iBut), *n*-hexane–*n*-heptane (Hex–Hept), benzene–toluene (BZN–TOL), *n*-heptane–toluene (Hept–TOL), cyclopentanol–cyclopentyl formate (CPOL–CPF), cyclohexanol–cyclohexyl formate (CHOL–CHF), methanol–tetrahydrofuran (MeOH–THF), α-pinene–limonene (Pin–Lim), diethylamine–water (DEA–H_2_O), formic acid–water (HCOOH–H_2_O), water–acetic acid (H_2_O–AcOH), oxygen–nitrogen (O_2_–N_2_), palmitic acid–stearic acid (Palm–Stear), formaldehyde–isoprenol (FA–Isop), and finally ethyl formate (HCOOEt) with *n*-alkanes from hexane to decane (Hex, Hept, Okt, Non, Dec).

The value displayed for the γ offset test is max­(|Δ*γ*_1_|, |Δ*γ*_2_|), and the consistency criterion Δ_max_ is set at 1.5%. Two columns are shown for both variants of the γ offset test: one using the ideal gas equation and the other using the virial equation. The value displayed for the Fredenslund test is $\max\left(\overset{―}{\delta p},\overset{―}{\Delta y_{1}},\overset{―}{\Delta y_{2}}\right)$ , as defined in eqs 12 and 13, and the consistency criterion is set at 1%,^5^ reflecting that none of the average residuals shall exceed 1%. The value displayed for the Redlich–Kister test is *D*, and the consistency criterion is set at 2 for isothermal data sets.^3^ For isobaric data, the consistency criterion is set at 10, making the test relatively permissive, which reflects its poor theoretical background for isobaric data.^9^ Values exceeding the consistency criteria are highlighted in red, indicating the rejection of thermodynamic consistency by the given testing procedure. The asterisk sign indicates that the *p* ^◦^ models used were those published or referenced in the same source. Otherwise, extended Antoine equation parameters were exported from the proprietary database of the Aspen Plus V14.5 software.^14^ These are expected to be relatively trustworthy because the software fits them to a large experimental database of vapor pressure data.

### General Observations

Table 2 shows some false positives of the *γ* offset test compared to the Fredenslund test, though only one false negative occurs when the ideal gas variant is used—the data set DEA–H_2_O “A”. More false negatives occur with the virial equation variant, namely, for the data sets Hept–TOL “A”, CPOL–CPF “C”, MeOH–THF “B”, and HCOOH–H_2_O “A”, At the selected Δ_max_ value of 1.5%, it is therefore confirmed quite well that rejection by the γ offset test (ideal gas variant) implies rejection by the Fredenslund test. A lower value would introduce false negatives and break the implication, while a higher value would decrease the test sensitivity by introducing more false positives. On its own, proving the validity of the implication does not justify the usefulness of the test, as it merely means that the test is weaker than that of Fredenslund. Instead, the value of the new test lies in its focused scope.

Meanwhile, the Redlich–Kister test is generally weaker for isobaric data than the new testing procedure due to its very permissive criterion of consistency. However, even so, several false negatives occur compared to the Fredenslund test. Indeed, lowering the criterion value for isobaric data would yield many more false negatives. We can therefore conclude that the Redlich–Kister test does not have added value when used together with the former two.

Naturally, there are cases of false positives, such as MeOH–H_2_O “C”, EtOH–H_2_O “A”, BZN–TOL “C”, CPOL–CPF “A” and “B”, and others, where the Fredenslund test detects thermodynamic inconsistency, but the γ offset test does not. These data sets likely contain errors, but primarily of a different kind than a mismatch between the VLE data and its corresponding *p* ^◦^ models. For example, the inconsistency could simply be caused by random error. When data with significant random error are fitted with a model, the model may extrapolate γ_*i*_(*x*_*i*_ = 1) very well to 1, allowing the *γ* offset test to pass. However, the average residuals of experimental values against model values will still be high, which leads to rejection by the Fredenslund test.

It should also be noted that the results of the *γ* offset test generally follow the same trend as the Fredenslund test results. Figure 3 plots the relationship of these results using an ideal gas variant of the *γ* offset test. For clarity, only cases without particularly glaring inconsistencies are displayed (<3%).

**Figure 3:**
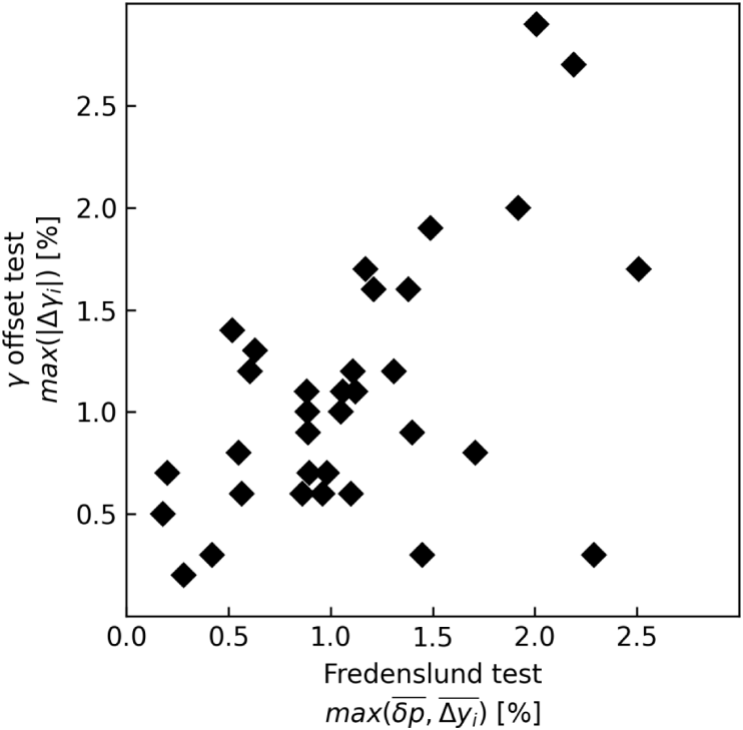
Plot of the γ offset test final result as the greater value of |*γ*_*i*_(*x*_*i*_ = 1) – 1| against the Fredenslund test final results, as the greatest value of $\overset{―}{\delta p}$ and $\overset{―}{\Delta y_{i}}$ .

Examining Table 2, let us first observe the differences between test results for the ideal gas and virial equation variants. Results almost always vary, but with several data sets, we may notice glaring discrepancies, a prime example being the data set Hept–TOL “E”. Looking at Figure 4, we can see that the data set shows significant random error, and model overfitting occurred. This is evidenced by Figure 5, which shows extremely high, physically unjustified values of the modeled fugacity coefficient. Constraints could be applied to the optimization procedure, but that would not solve the core issue—the combined model, which describes both gas- and liquid-phase nonideality, offers too many degrees of freedom for such a data set. The virial gas variant is therefore not suitable for data with significant random error, and the ideal gas variant should be preferred. Though it must be emphasized that at its core, the proposed testing procedure provides an empirical numerical comparison, with reliable fitting and extrapolation being the main goal. Physically meaningful *ϕ* values are not to be expected in any case but should contribute to a good fit. The data sets MeOH–H_2_O “B”, “D”, CPOL–CPF “C”, and BZN–TOL “C” present a similar case. The data set Palm–Stear “A” could not be successfully fitted with the virial equation model due to its random error. Meanwhile, the virial equation fitting was not performed with data sets Hept–TOL “B” and HCOOH–H_2_O “D” due to an insufficient number of data points.

**Figure 4:**
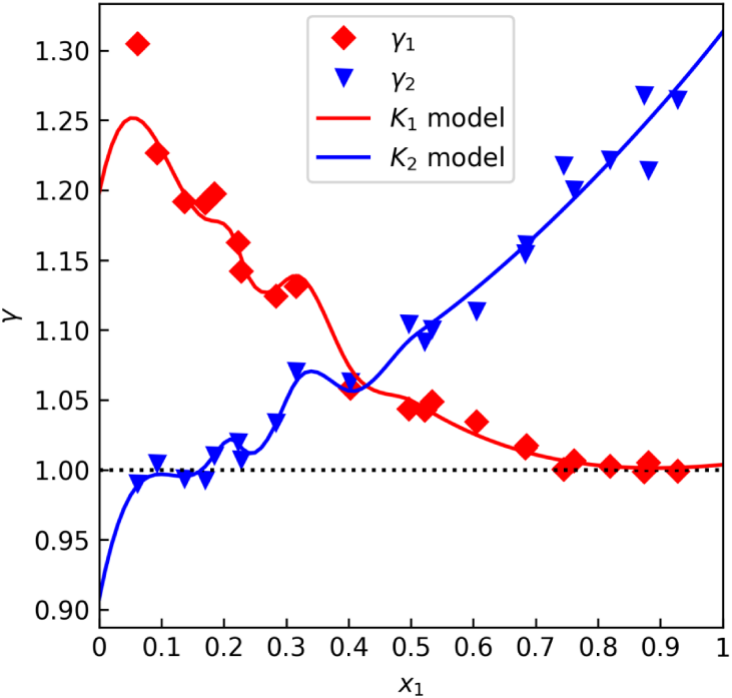
A case of the γ offset test virial equation overfitting for the heptane–toluene data set “E” by L. Sieg^48^ (isobaric at 101 kPa).

**Figure 5:**
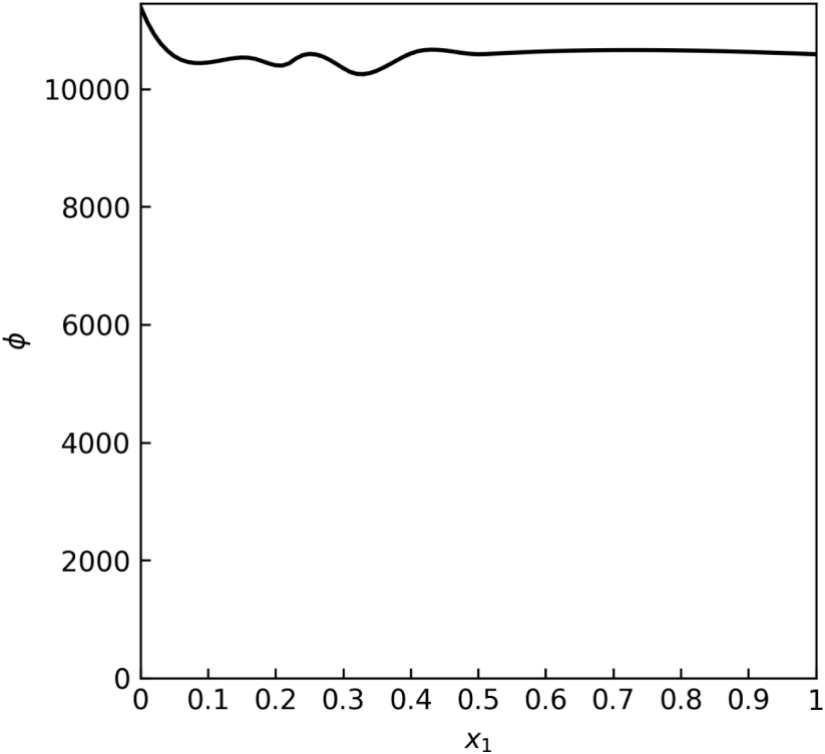
A case of the *γ* offset test virial equation overfitting for the fugacity coefficient model for the heptane–toluene data set “E” by L. Sieg^48^ (isobaric at 101 kPa).

An important aspect of the proposed test is its ability to provide partial results. The data set HCOOH–H_2_O “A” covers a range of compositions only up to 68% HCOOH. Figure 6 shows the test results for the virial equation variant. Although the ideal gas variant result was below the consistency criterion, we may attribute this to chance, as the extrapolation will not be reliable at *x*_1_ = 1. However, the test results are very favorable at *x*_2_ = 1, yielding |Δ*γ*_2_| = 0.9% with the ideal gas variant and |Δ*γ*_2_| = 0.3% with the virial equation variant. Table 2 lists only the greater value for brevity, but we can see that for data with a limited composition range, it may be useful to discard one of the results and consider the consistency of only one of the two $p_{i}^{◦}$ models.

**Figure 6:**
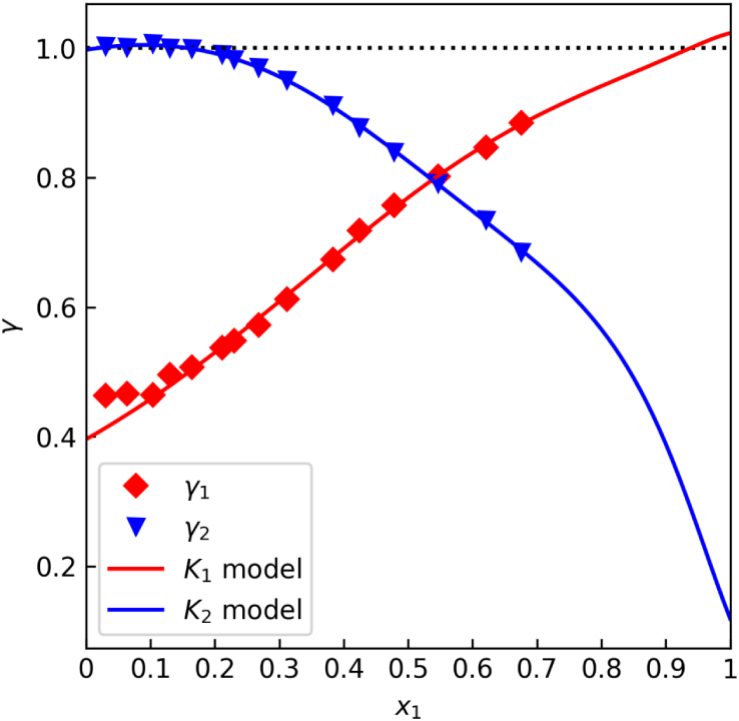
Partially interpretable *γ* offset test for the formic acid–water data set “A” by Conti et al.^56^ (isobaric at 101.3 kPa).

### Case Studies

When we closely examine the EtOH–H_2_O data set “B”, we may notice different results depending on the source of the *p* ^◦^ models. In this case, the models from the Aspen Plus V14.5 software yield more favorable results than the models cited by the authors of the data. We may assert that the published VLE data set is more consistent with the former than with the latter. Using the authors’ models alongside the VLE data would further contribute to the already significant systematic error. Without any external reference, the test can evaluate the internal consistency of the VLE data and *p* ^◦^ models—but not their absolute quality.

Looking at the results for hydrocarbon systems, namely, hexane–heptane (Hex–Hept), benzene–toluene (BZN–TOL), and heptane–toluene (Hept–TOL), it is striking that the Redlich–Kister test yields significantly higher results, even when both other tests agree on thermodynamic consistency. A possible explanation lies in the fact that these systems exhibit lower activity coefficients compared to those of other systems. For these almost ideal mixtures, $\ln\frac{\gamma_{1}}{\gamma_{2}}$ is very close to zero within the whole composition range. Therefore, even a minute experimental error can systematically shift it above or below zero. That strongly affects the relative areas calculated by integration—indeed, for some data sets, the calculated $\ln\frac{\gamma_{1}}{\gamma_{2}}$ value does not ever cross zero, yielding *D* = 100. A thorough analysis of the Redlich–Kister test lies beyond the scope of this article, but suffice it to say that the proposed test does not exhibit the same error for a system closer to ideal behavior.

Not much data are available in the literature for the systems methanol–tetrahydrofuran (MeOH–THF) and α-pinene–limonene (Pin–Lim), but in both cases, we can see a clear distinction between a set of thermodynamically consistent data “B” and inconsistent data “A”. Note that using the *p* ^◦^ model from Aspen Plus does not alleviate the issue, which excludes the *p* ^◦^ model referenced by the authors as the sole source of error. The new test therefore hints that it is the VLE data set that should be revised.

The system diethylamine–water (DEA–H_2_O) is an example of a system with exceptionally strong liquid-phase nonideality, as evidenced by the data set “C”. This is illustrated in Figure 7, where we may observe that the NRTL and virial equation models fit the data well, despite activity coefficients reaching up to 28 at one data point.

**Figure 7:**
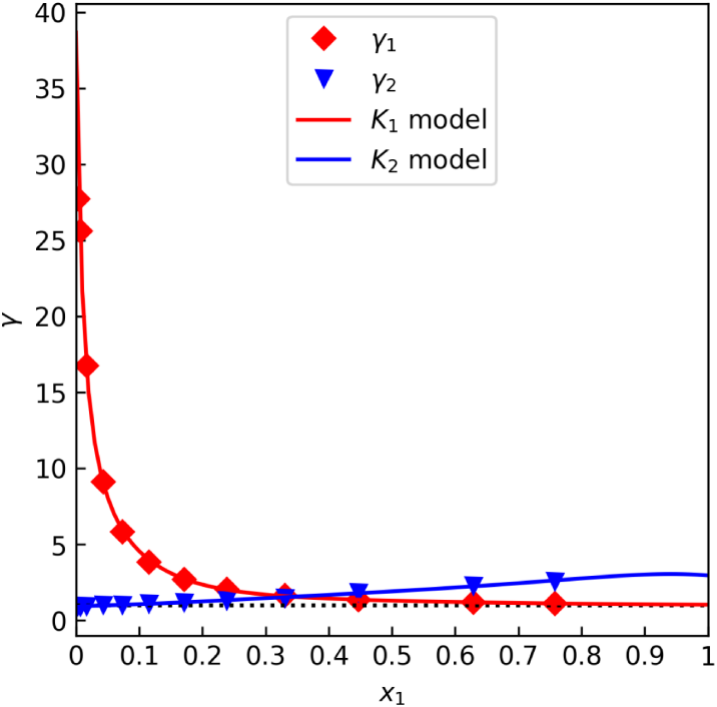
γ offset test for the diethanolamine–water data set “C” by Frangieh et al.^55^ (isobaric at 101.3 kPa).

The systems formic acid–water (HCOOH–H_2_O) and water–acetic acid (H_2_O–AcOH) are of particular interest due to their strong vapor-phase association caused by hydrogen bonds, even at lower pressures.^19,20^ Considering this fact, the virial equation variant should be preferred over the ideal gas variant. Since the traditional tests do not take gas-phase nonideality into account, it should come as no surprise that they yield very high test results. Those results should be considered with caution, as they may be less reliable for such a system. The same can be said for the systems propane–isobutane (Prop–iBut) and oxygen–nitrogen (O_2_–N_2_), which are also unlikely to comply with assumed ideal behavior in the vapor phase. In these cases, the reason lies in the immensely high pressures and cryogenic temperatures needed to liquefy these gases. Among the studied data sets, the highest pressure reached 2.2 MPa in Prop–iBut “B”, while the temperature was maintained as low as 100 K in O_2_–N_2_ “A”. Arguably, this new test is particularly valuable for such systems. In the case of data sets H_2_O–AcOH “B”, “C” and Prop–iBut “A”, “B”, the virial equation variant yields lower test results. Considering that gas-phase nonideality is expected, it remains a possibility for these cases that the systematic error is of a different kind than the one detected by the new test, and the ideal gas variant reports a false negative. For such systems, it is therefore suitable to always apply both test variants and discuss the results individually.

Last but not least, the systems of ethyl formate with various *n*-alkanes demonstrate a general trend of increasing thermodynamic inconsistency with increasing relative volatility. Indeed, the vapor-phase composition approaches the pure component for systems with high relative volatility. Commonly used analytical methods may become very sensitive to experimental error when any of the *x*_i_ ≈ 0. More homologous series of systems would need to be researched to explore this observation, but let us remark that the proposed test follows the same trend as other tests to some degree. The new test is therefore equally unsuitable for systems with very high relative volatility.

### Application on Reactive Distillation

The system formaldehyde–isoprenol, as studied by M. Dyga et al.,^70^ stands out in Table 2 with its particularly high results for the γ offset and Redlich–Kister tests. Intense formaldehyde oligomerization was reported for this system, which explains why it exhibits extreme deviations from the behavior expected by tests of thermodynamic consistency. None of the discussed or proposed test procedures take chemical reactions into account, making them insufficient to confirm or reject thermodynamic consistency.

Nevertheless, a detailed examination of the new γ offset test shows that it can still offer limited informational value. While the tests of Fredenslund and Redlich–Kister both resolutely reject the data, the γ offset test has the advantage of providing two component-wise result values, instead of one or more values that represent the whole data set equally. For brevity, Table 2 shows only the larger of the two |Δ­{*γ*_*i*_| values. In both cases, it was |Δ*γ*_1_(*x*_1_ = 1)| that reached almost 100%, demonstrating that the extrapolated behavior of near-pure formaldehyde is far from the expected volatility of a pure nonreactive component. It should be noted that the data covers only the region poor in formaldehyde (*x*_1_ < 0.4), making this extrapolation unreliable. The limited composition range may also be the reason why the virial equation optimization did not successfully converge, so only the ideal gas variant was used.

However, in the near-pure isoprenol region, the results were |Δ*γ*_2_(*x*_2_ = 1)| = 1.3% for the data set “A” and |Δ*γ*_2_(*x*_2_ = 1)| = 0.3% for “B”, as illustrated in Figure 8. The test has proven that, at least in the near-pure isoprenol region, both VLE data sets are very consistent with the *p* ^◦^ model referenced by the authors. A partial verdict on thermodynamic consistency is a novel contribution for a system that would otherwise be untestable. We may conclude that the *γ* offset test is superior for binary systems where only one of the constituent components is readily reactive.

**Figure 8:**
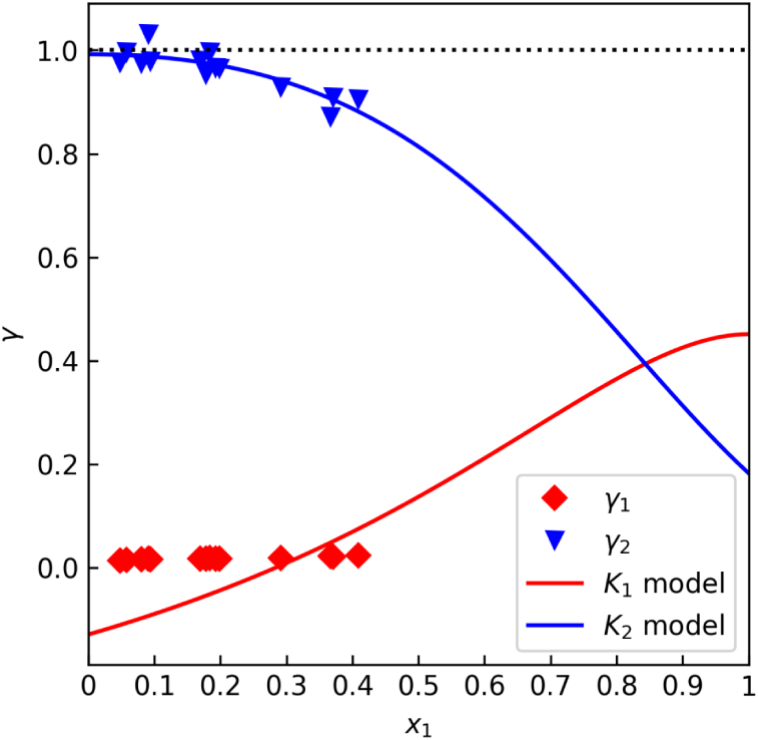
*γ* offset test for the formaldehyde–isoprenol data set “B” by M. Dyga et al.^70^ (isothermal at 393.1 K).

### Comparison of Discussed Test Procedures

An overview of the discussed testing procedures is provided in Table 3. The Herrington test, the point-to-point test of van Ness, and the slope test are also included for reference.

**Table 3:** Overview of Tests of Thermodynamic Consistency for Binary VLE Data Sets^a^

Testing procedure	Data set summary metric	Individual point metric	Requires whole *x*_1_ range	Isobaric suitability	Isothermal suitability	Minimal data size^b^
Fredenslund^5^	Yes	Yes	Yes	Yes	Yes	5
γ offset	Yes	No	Partially^c^	Yes	Yes	5
Redlich–Kister^3^	Yes	No	Yes	N	Approx.	2
Herington^4^	Yes	No	Yes	Approx.	No	2
Van Ness^6^	Yes	Yes	No	Yes	Yes	1
Slope^9^	No	Yes	No	Yes	Yes	2

a Approx. means that the procedure is an approximation but may be used with consideration.

b The bare minimum to technically perform the procedure, but more data points may be required for reliable results.

c Requires at least one of the near-pure regions to be covered by data to give a reliable partial result.

## Conclusions

A new test of VLE thermodynamic consistency, called the “gamma offset test”, was proposed in this article to be used on binary VLE data sets. The test is based on the NRTL model and can be used in two variants: one with the ideal gas model and the other with the first-order virial equation of state. The model parameters are optimized using nonlinear regression, with the total number of parameters ranging from 4 to 9, depending on the configuration. The procedure is therefore designed to be performed on a data set with 5 or more points. Unlike traditional testing procedures, this test is not based on the Gibbs–Duhem equation but rather on compliance with the definition of activity as such. It was then tested on a number of isobaric or isothermal data sets from the literature. The results were compared with those of the widely used tests of Fredenslund and Redlich–Kister. The new test was designed to detect and quantify a specific source of experimental error—the inconsistency between the VLE data set and the corresponding vapor pressure models for both pure components. For instance, the test was not designed to detect random error as a source of error, like the test of Fredenslund. The test yields two |Δ*γ*_*i*_| result values, defined as an extrapolation |*γ*_*i*_(*x*_*i*_ = 1) – 1|. A criterion of consistency Δ_max_ was set at 1.5% to formally accept or reject the thermodynamic consistency based on the higher value of the two |Δγ_*i*_|. It was confirmed that with this criterion value, rejection of consistency by the γ offset test using the ideal gas variant implies rejection by the Fredenslund test, which was expected due to the focused scope of the test. The ideal gas variant was found to be mathematically stable and universally applicable. Meanwhile, the virial equation variant remains a specialized tool for systems where strong gas-phase nonideality is expected but may be less stable. Particularly if the data set is small or exhibits significant random error, overfitting has proven to be a problem when optimizing the model with the virial equation. We therefore recommend performing both variants of the test for comparison. Case studies of test applications were used to show how the results can be meaningfully interpreted and how the new test can serve to pinpoint the source of thermodynamic inconsistency within the data set. Moreover, the new test provides two separate results for both constituent components of the binary system, which proved to be advantageous for systems with a limited concentration range or highly reactive systems. In these cases, it offers at least a partial verdict on thermodynamic consistency by considering only one of the two |Δ*γ*_*i*_| values. The test was applied to a broad range of systems and was demonstrated to be suitable even for systems with strong gas-phase nonideality or particularly strong nonideal behavior in the liquid phase.

No added value was found in carrying out the Redlich–Kister test alongside the former two. The Redlich–Kister test was found to be overall less sensitive compared to the Fredenslund test, yielding many false positives without providing the focused, detailed results of the newly proposed test. On the other hand, it also proved to be excessively sensitive for almost ideal systems.

The *γ* offset test is part of a new software package called “VLizard, a VLE wizard”. This freely available open-source software^21^ was introduced with the hope of becoming a valuable tool for both academic and industrial research on vapor–liquid equilibria. The application implements the new procedure, as well as several well-known and widely used testing procedures. It also features nonlinear regression for both vapor pressure and binary VLE data. Its graphical interface is designed to be easily accessible to experimental researchers without any programming background. This software may be used both for academic research as well as for the research and design of industrial separation processes, where the quality of the VLE data is of utmost importance.

## Supplementary Material





## Notes

Table S1: Fredenslund test summarized results; Figure S1: Fredenslund test *g*_E_ visualization for methanol–water data sets “A” by Kurihara et al.^31^ and “B” by Bredig et al.;^32^ Figure S2: Fredenslund test *δ_p_* visualization for methanol–water data sets “A” by Kurihara et al.^31^ and “B” by Bredig et al.;^32^ Figure S3: Fredenslund test Δ*y_i_* visualization for methanol–water data sets “A” by Kurihara et al.^31^ and “B” by Bredig et al.;^32^ Table S2: area test results applicable for Redlich–Kister and Herington tests; Figure S4: area test visualization for methanol–water data sets “A” by Kurihara et al.^31^ and “B” by Bredig et al.;^32^ Figure S5: slope test residuals visualization for methanol–water data sets “A” by Kurihara et al.^31^ and “B” by Bredig et al.,^32^ with y-axes displaying the *r*_*j*_ defined in eq 16; Table S3: optimized NRTL parameters and optimization metrics for the two methanol–water data sets; Table S4: van Ness point-to-point test results using the respective NRTL model for each source as per Table S3; Figure S6: visualization of activity coefficient NRTL fitting for methanol–water data sets “A” by Kurihara et al.^31^ and “B” by Bredig et al.;^32^ Figure S7: van Ness point-to-point test visualization for methanol–water data sets “A” by Kurihara et al.^31^ and “B” by Bredig et al.,^32^ with *y*-axis showing the $\ln\frac{\gamma_{1}}{\gamma_{2}}$ residuals between the experimental data and the NRTL model as per Table S3 (PDF)
“Vapor-liquid equilibrium data sets.csv”, a file with all the VLE data sets referenced in Table 2 (CSV)

## Abbreviations

VLE: vapor–liquid equilibrium
UNIQUAC: universal quasi-chemical activity coefficient
MeOH: methanol
EtOH: ethanol
Prop: propane
iBut: isobutane
Hex: hexane
Hept: heptane
Oct: octane
Non: nonane
Dec: decane
BZN: benzene
TOL: toluene
CPOL: cyclopentanol
CPF: cyclopentyl formate
CHOL: cyclohexanol
CHF: cyclohexyl formate
THF: tetrahydrofuran
DEA: diethylamine
AcOH: acetic acid
HCOOH: formic acid
HCOOEt: ethyl formate
Pin: α-pinene
Lim: limonene
Palm: palmitic acid
Stear: stearic acid
FA: formaldehyde
Isop: isoprenol

## Symbols

Δ: absolute deviation
δ: relative deviation
*p*: pressure
*T*: temperature
*x*_*i*_: mole fraction of component *i* in the liquid phase
*y*_*i*_: mole fraction of component *i* in the vapor phase
*γ*_*i*_: activity coefficient of component *i*
$p_{i}^{◦}$: vapor pressure of pure component *i*
*ϕ*: fugacity coefficient of the gas phase as a single pseudocomponent
$\phi_{i}^{◦}$: fugacity coefficient of pure component *i* saturated vapors
*K*_*i*_: overall nonideality factor for component *i*
*Z*: gas compressibility factor
*R*: universal gas constant, considered to be 8.31446 J/(K mol)
*B*: first virial coefficient of the gas phase as a single pseudocomponent
*B*_*i*_: _*i*_first virial coefficient of pure component *i*
*B*_12_: first virial symmetrical cross coefficient of a binary mixture
*a*_*ij*_,*b*_*ij*_,*c*_12_: NRTL model binary interaction parameters
*E*_1_,*E*_2_: modified NRTL model error parameters
*r*_*i,j*_: residual of *i*-th component for *j*-th data point
|Δ*γ*_*i*_|: “gamma offset”, defined as extrapolation |*γ*_*i*_(*x*_*i*_ = 1) – 1|
Δ_max_: “gamma offset test” criterion of consistency
Δ_exp_ *f*: *f* experimental uncertainty of a quantity *f*
*A*,*B*,*C*,*D*,*E*,*F*,*G*: extended Antoine equation parameters
*n*_L_: maximum order of the sum of Legendre polynomials
$\overset{―}{\delta p}$: average relative deviation of pressure
$\overset{―}{\Delta y_{i}}$: average absolute deviation of the vapor-phase mole fraction of component *i*
*D*: metric for both area tests
*J*: Herington test metric
