# Supplementary material for: A New Method for Testing Thermodynamic Consistency of Vapor–Liquid Equilibrium Data
Source: ACS Omega. 2025 Oct 2;10(40):46809–21. doi: 10.1021/acsomega.5c04650 (PMC12529394; doi:10.1021/acsomega.5c04650)
Supplement: Supplementary file 1 [file ao5c04650_si_001.pdf]

A new method for testing thermodynamic consistency of vapor-liquid equilibrium data

Authors

Jiří Zbytovský (Jiri.Zbytovsky@vscht.cz), Tomáš Sommer (Tomas.Sommer@vscht.cz), Martin Zapletal (Martin.Zapletal@vscht.cz) and Jiří Trejbal (Jiri.Trejbal@vscht.cz))*

* Corresponding author
Email: Jiri.Zbytovsky@vscht.cz
Department of Organic Technology, University of Chemistry and Technology Prague, Technická 5, 166 28 Prague 6, Czech Republic

Author affiliations

- Jiří Zbytovský: Department of Organic Technology, UCT Prague
- Tomáš Sommer: Department of Organic Technology, UCT Prague
- Martin Zapletal: Department of Organic Technology, UCT Prague
- Jiří Trejbal: Department of Organic Technology, UCT Prague

Abstract

Tests of thermodynamic consistency are an essential tool to evaluate VLE data quality. However, there is a lack of software that would offer the most commonly used testing procedures in one application. Furthermore, currently used tests are very general and serve well to quantify the experimental error, but do not reveal much about its cause. In this work, a new test is proposed, called the “gamma offset test”. It is designed to have a focused, limited scope – to detect inconsistency between the binary VLE data set and the corresponding vapor pressure models. The proposed testing procedure was applied on a collection of VLE data sets obtained from literature, and the results were compared with tests of Fredenslund and Redlich-Kister. A criterion of consistency to formally accept or reject the data was fine-tuned, so that the test provides meaningful results. It was shown that the new test can be a valuable complement to the traditionally used procedures for most binary systems. Moreover, advantages of the new test were demonstrated at systems where existing procedures are difficult to apply. The new test can be used either to assess experimental setup accuracy, or to help diagnose the cause of a known experimental error. This test is part of a newly developed free open-source software package called “VLizard, a VLE wizard”, which also offers other well-known testing procedures. With its graphical interface it aims to fill the gap as an easily accessible tool for both academic and industrial VLE research.

Keywords

Thermodynamic consistency; VLE; Software; Herington test; Redlich-Kister test; Van Ness test; Fredenslund test;

Supporting information

Fredenslund test results

Table S1: Fredenslund test summarized results.

Source	$\overline{\delta p}$	$\overline{\Delta y_1}$	$\overline{\Delta y_2}$
A [1]	0.77 %	0.96 %	0.20 %
B [2]	3.55 %	4.67 %	1.32 %

Legendre polynomial order was set $n_\mathrm{L} = 4$. $\overline{\delta p}$ and $\overline{\Delta y_i}$ are defined in equations 12 and 13 and the consistency criterion is 1 % for all of them.

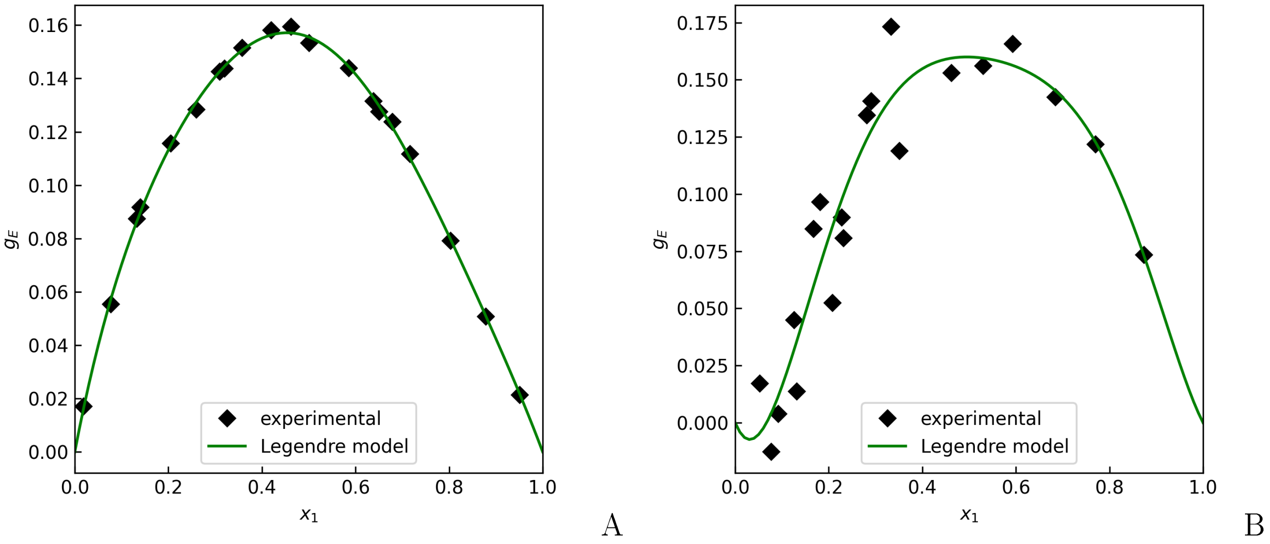

Figure S1: Fredenslund test g_E visualization for methanol–water data sets “A” by Kurihara et al. [1] and “B” by Bredig et al. [2].

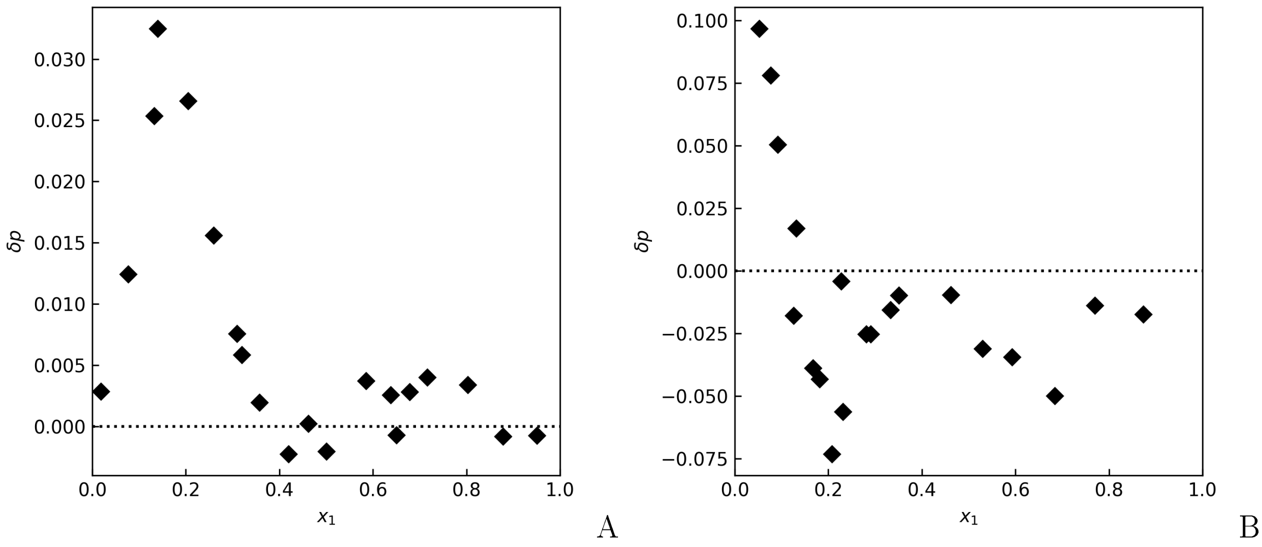

Figure S2: Fredenslund test δp visualization for methanol–water data sets “A” by Kurihara et al. [1] and “B” by Bredig et al. [2].

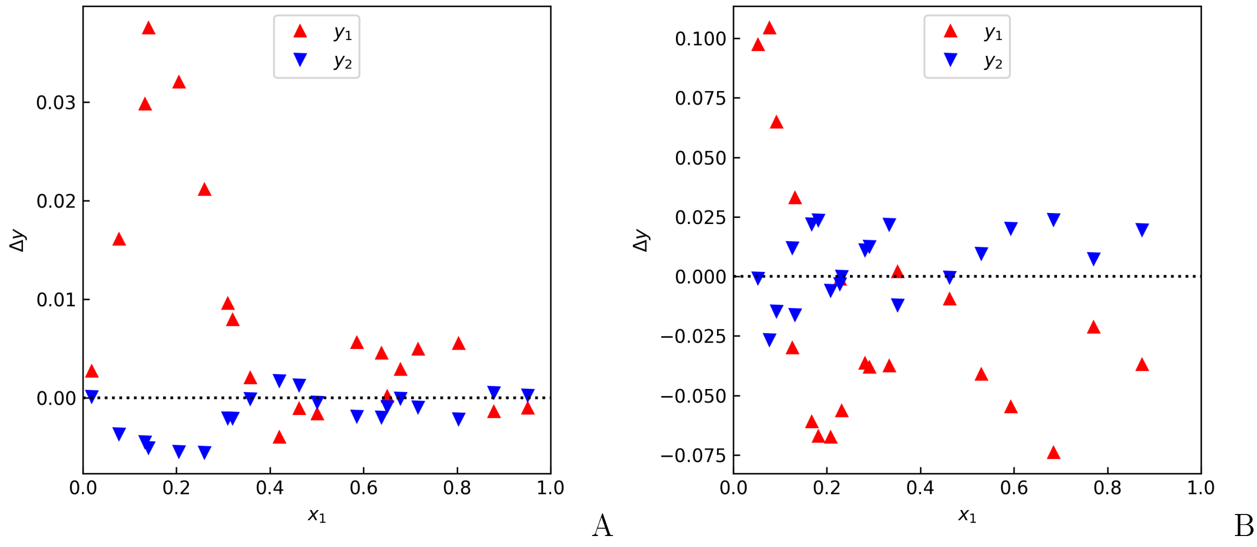

Figure S3: Fredenslund test Δy_i visualization for methanol–water data sets “A” by Kurihara et al. [1] and “B” by Bredig et al. [2].

Area tests results

Table S2: Area test results applicable for Redlich-Kister and Herington tests.

Source	D	$a-b$	$a+b$	J	$\|D-J\|$
A [1]	5.74	0.0188	0.328	13.8	8.04
B [2]	16.0	0.0792	0.493	11.5	4.58

D and J are defined in the equations 14 and 15. a and b are the areas delimited by the $\ln \frac{\gamma_1}{\gamma_2}$ curve. Both data sets are isobaric, the consistency criterion for the Redlich-Kister test is $D < 10$ and for the Herington test it is $|D-J| < 10$.

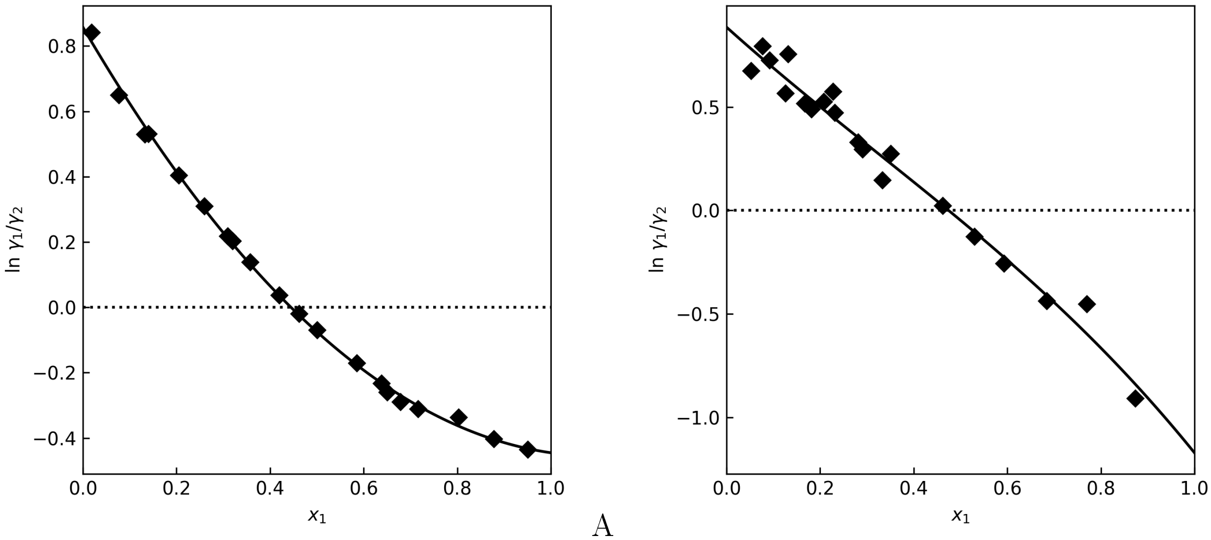

A B

Figure S4: Area test visualization for methanol–water data sets “A” by Kurihara et al. [1] and “B” by Bredig et al. [2].

Slope test results

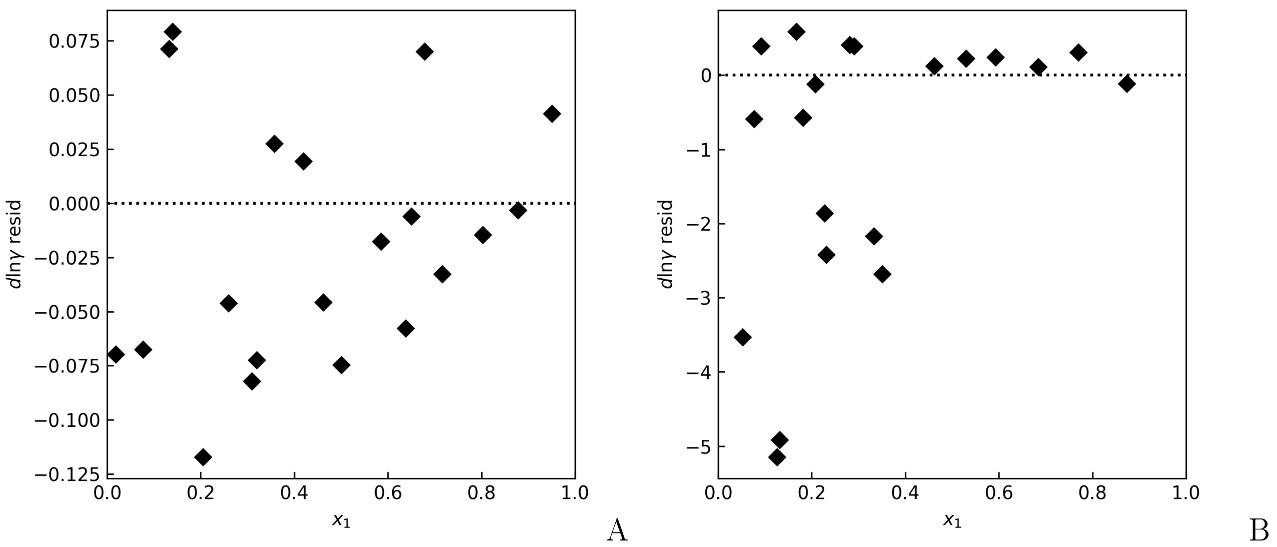

Figure S5: Slope test residuals visualization for methanol–water data sets "A" by Kurihara et al. [1] and "B" by Bredig et al. [2], with y-axes displaying the r_j defined in equation 16.

van Ness test with NRTL fitting results

Table S3: Optimized NRTL parameters and optimization metrics for the two methanol–water data sets.

Source	a_{12}	a_{21}	b_{12}	b_{21}	c_{12}	RMS	AAD
A [1]	1.414	0.524	-590.9	203.8	0.3	0.014	0.011
B [2]	-10.31	7.957	3666	-2508	0.3	0.093	0.077

The parameter c_{12} was kept constant. The RMS (root mean square) and AAD (average absolute deviation) are calculated from serialized γ_1 and γ_2 values.

Table S4: van Ness point-to-point test results using the respective NRTL model for each source as per Table S3.

Source	Residuals root mean square	Consistency index as $\left\lceil \frac{RMS}{2.5\%} \right\rceil$
A [1]	0.018	1
B [2]	0.089	4

The root mean square is calculated from $\ln \frac{\gamma_1}{\gamma_2}$ residuals between the experimental data and the NRTL model as per Table S3.

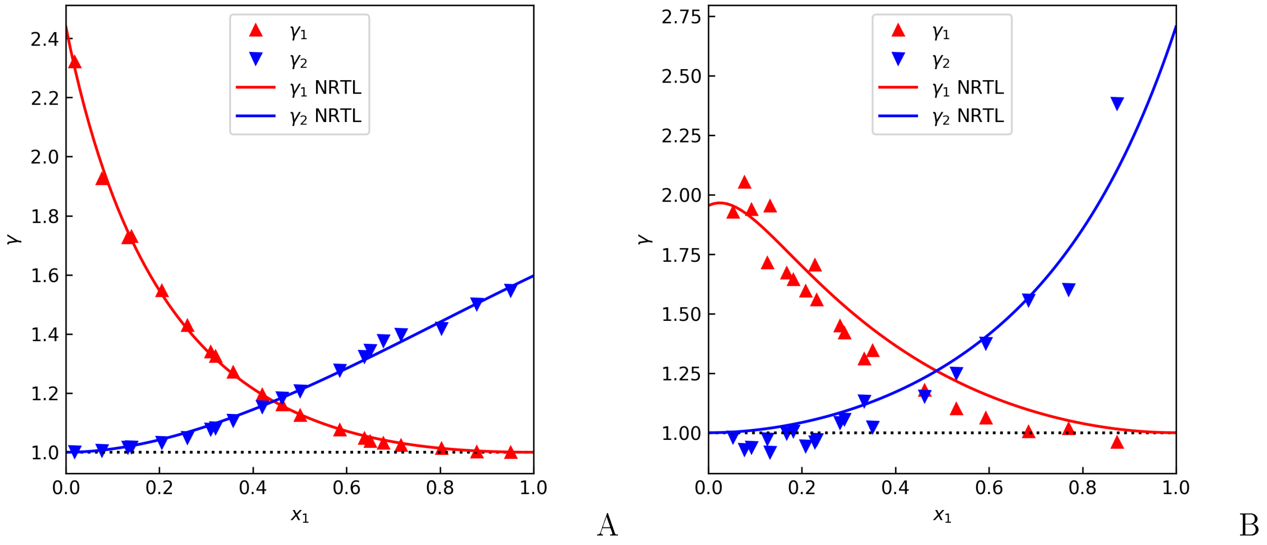

Figure S6: Visualization of activity coefficient NRTL fitting for methanol–water data sets "A" by Kurihara et al. [1] and "B" by Bredig et al. [2].

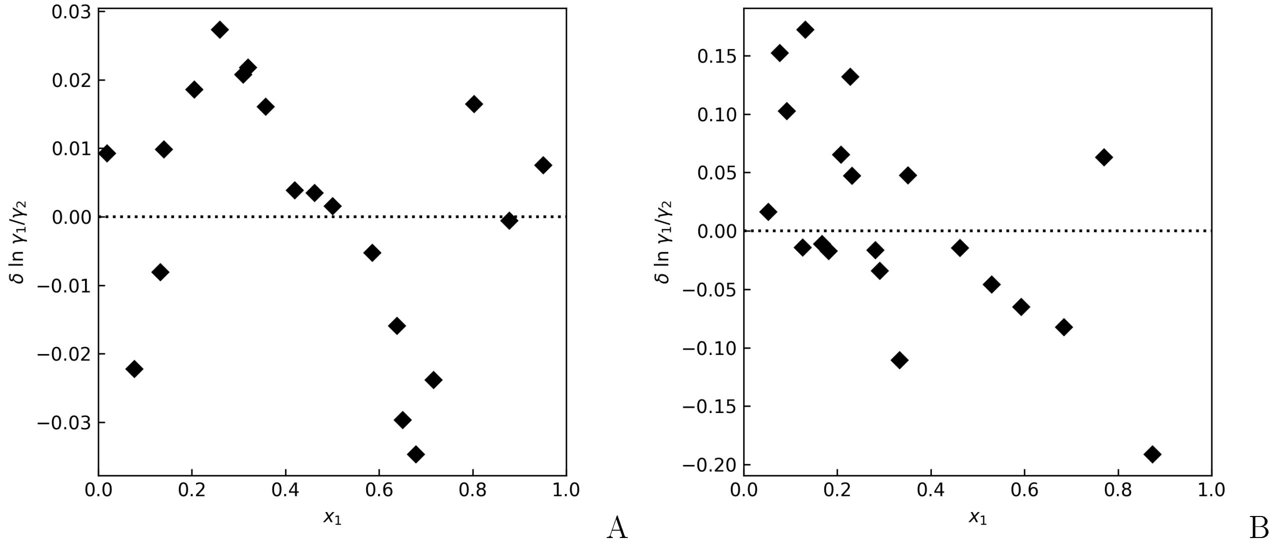

Figure S7: van Ness point-to-point test visualization for methanol–water data sets "A" by Kurihara et al. [1] and "B" by Bredig et al. [2], with y-axis showing the $\ln \frac{\gamma_1}{\gamma_2}$ residuals betwen the experimental data and the NRTL model as per Table S3.

Table S5: Methanol–water VLE data by Kurihara et al. [1], labeled “A”.

p/kPa	T/K	x_1	y_1
101.3	369.56	0.019	0.137
101.3	362.72	0.077	0.368
101.3	358.38	0.133	0.492
101.3	357.76	0.140	0.508
101.3	354.51	0.205	0.594
101.3	352.45	0.260	0.647
101.3	350.86	0.310	0.683
101.3	350.58	0.320	0.690
101.3	349.54	0.358	0.713
101.3	348.07	0.420	0.746
101.3	347.09	0.462	0.768
101.3	346.38	0.501	0.787
101.3	344.70	0.586	0.826
101.3	343.83	0.638	0.848
101.3	343.69	0.651	0.852
101.3	343.11	0.679	0.864
101.3	342.41	0.717	0.882
101.3	340.89	0.803	0.922
101.3	339.66	0.879	0.952
101.3	338.46	0.951	0.981

Table S6: Methanol–water VLE data by Bredig et al. [2], labeled "B".

p/kPa	T/K	x_1	y_1
101.32	366.02	0.0531	0.2834
101.32	363.42	0.0767	0.4001
101.32	362.02	0.0926	0.4353
101.32	359.72	0.1257	0.4831
101.32	358.12	0.1315	0.5455
101.32	356.32	0.1674	0.5585
101.32	355.42	0.1818	0.5775
101.32	354.72	0.2083	0.6273
101.32	352.12	0.2276	0.6674
101.32	353.32	0.2319	0.6485
101.32	351.12	0.2818	0.6775
101.32	350.92	0.2909	0.6801
101.32	349.82	0.3333	0.6918
101.32	349.32	0.3513	0.7347
101.32	346.92	0.4620	0.7756
101.32	345.82	0.5298	0.7971
101.32	344.42	0.5937	0.8183
101.32	343.12	0.6849	0.8492
101.32	341.12	0.7701	0.8962
101.32	340.02	0.8741	0.9194

References

1. Kurihara, K.; Nakamichi, M.; Kojima, K. Isobaric vapor-liquid equilibria for methanol+ ethanol+ water and the three constituent binary systems. *Journal of Chemical and Engineering Data* **1993**, *38*, 446–449.

2. Bredig, G.; Bayer, R. II. Vapor pressure of the ternary system methanol-methyl acetate-ethyl acetate. *Z. Phys. Chem. Stoechiom. Verwandtschaftsl.* **1927**, *130*, 15–28.
